# Determining the appropriate concentration of an anesthetic mixture in three different fish species with the PROMETHEE decision model

**DOI:** 10.3389/fvets.2024.1492769

**Published:** 2024-11-06

**Authors:** Mert Minaz, Akif Er, Kübra Ak, İlker Zeki Kurtoğlu, Şevki Kayış

**Affiliations:** Department of Aquaculture, Faculty of Fisheries, Recep Tayyip Erdoğan University, Rize, Türkiye

**Keywords:** anesthesia, common carp, Danube sturgeon, herbal anesthetics, multi-criteria decision model (MCDM), rainbow trout

## Abstract

Anesthesia is applied to protect fish welfare for reducing employee workload in aquaculture. The efficacy of the anesthetic agent varies depending on the fish species. In this study, the effect of a commercial anesthetic (VetiVital AquaSED) manufactured mainly with herbal extracts (includes eugenol, linalool, linalyl acetat etc.) on three different fish species (rainbow trout, common carp, and Danube sturgeon) was investigated. In addition, the best available concentration of the anesthetic mixture for each species was determined using the PROMETHEE decision model. Danube sturgeon showed more resistance to anesthetics than the other two species. It was determined that the increase in concentration caused histological deterioration in fish. On the other hand, hematological parameters were affected by the anesthetic mixture, and this effect returned to normal levels after 8 h. According to the multi-criteria decision model, the best available concentrations determined by considering 10 different criteria are 400, 480, and 675 mg L^−1^ for rainbow trout, common carp and Danube sturgeon, respectively. Future studies should focus on developing the most appropriate anesthesia mixture in terms of physiological and anesthesia effectiveness for the target fish species.

## 1 Introduction

Routine operational processes such as vaccination, handling and transportation in aquaculture cause a stressful environment that compromises animal welfare (1). Ultimately, as the fish’s response to stressors, resistance to diseases decreases and physiological disruptions occur (2). In order to eliminate such negative effects in aquaculture, anesthetic substances are used before any operation to fish (3). Anesthetic agents are used in a wide range of applications, from light sedation to reduce stress to full anesthesia to eliminate pain during surgery (4). This is an ethical and scientific obligation as well as a legal obligation (5). For this purpose, various synthetic [tricaine methanesulfonate (MS-222), benzocaine, 2-phenoxyethanol etc.] and natural anesthetic (herbal essential oils) agents are used in aquaculture (6). Although MS-222 is the only approved anesthetic agent, it has some disadvantages such as inducing hyperglycemia and oxidative stress (7). The potential adverse effects of synthetic anesthetic agents on the environment and organisms have triggered the search for alternative anesthetics of herbal origin (8). The sedative effects of various herbal essential oils on fish have been previously investigated (9–13). Commercial anesthetic mixtures prepared with plant essential oils that can replace synthetic anesthetics also need to be developed.

The effectiveness of an anesthetic agent is evaluated by many factors such as induction time on the organism, physiological effects and cost analysis (14, 15). The physiological, histological, oxidative and hematological effects of a newly applied anesthetic agent on fish provide an idea in terms of welfare and health. Since each species has its own physiological and behavioral responses, it would not be correct to generalize all fish to anesthetic agents. In this context, it is a correct approach to evaluate different species, especially when using agents that have not been researched before. Due to differences in physiological demands, survival standards vary according to the trophic level of the fish. Each organism’s response to any anesthetic agent is different and therefore the effectiveness of the anesthetic agent varies from fish to fish (8). In this context, it is necessary to perform multivariate modeling with different types of fish.

Common carp (*Cyprinus carpio*), one of the fish with the largest share in the aquaculture sector, is a warm water fish with a low trophic level. It is a potential candidate for aquaculture with its high adaptability to environmental conditions (16). Common carp accounts for more than 80% of total fish production in many European countries (17). On the other hand, Danube sturgeon (*Acipenser gueldenstaedtii*) is an important fish species with high potential for world aquaculture due to its high resistance to environmental mechanisms and high economic added value (commercial value of its meat and caviar) (18). Additionally, Danube sturgeon is one of the vulnarable fish species in the Red List of Threatened Species by IUCN (19). Therefore, it is important to use the most appropriate anesthetic agent for the fish in all cultivation operations. And, the rainbow trout (*Oncorhynchus mykiss*), which lives at a higher trophic level than the other two species, is the one of the most cultured cold-water fish in the world with its significant market value and meat quality (20). Rainbow trout, a fish that is very sensitive to environmental conditions such as temperature, dissolved oxygen (DO), and organic nutrients, is subjected to sedation or deep anesthesia during operational processes. When all these species are evaluated together due to their different trophic levels and possible different responses to anesthetic agents, they will reveal the overall effect of the target anesthetic agent. In this context, the current study focused on the physiological effect of a new commercial anesthetic on fish species belonging to three different trophic levels. The specific objectives of the current study are:

- to expose three different fish species to a commercial anesthetic at low, medium and high concentrations,
- to determine the induction and recovery times of fish for each concentration,
- to observe the histological and hematological responses of fish to each concentration, and
- to determine appropriate anesthetic mixture concentrations for the target fish species by the Prevent Ranking Organization Method for Enrichment (PROMETHEE).

## 2 Materials and methods

### 2.1 Anesthetic mixture

The anesthetic mixture has been commercially produced (VetiVital AquaSED, İzmir, Türkiye). It contains a naturally defined aromatic plant mixture (2b 906.250 mg) and sorbitan monooleate (E494) and guar gum (E412) as emulsifiers. Anesthetic mixture is a water-soluble liquid material with a white color and aromatic odor. Composition of anesthetic mixture is presented in Table 1. Analyses were per formed on a GC–MS (Shimadzu QP2010 Ultra) device with a 30 m 5-Ms column. The transfer line and ion source temperatures were set at 280 and 275°C, respectively. Qualitative analysis was performed utilizing the NIST and Wiley libraries that are integrated into the device. The GC–MS analysis was conducted as an outsourced service at the Central Research Laboratory Application and Research Center at Recep Tayyip Erdoğan University. Qualitative analysis was done using NIST and Wiley libraries integrated in the device. Accordingly, eugenol, linalyl acetate, 1-Propanol, 2-ethoxy- and linalool showed the highest area and height, respectively. Based on the package insert, the estimated application concentration is 80–100 mg L^−1^. The suitability of the commercial anesthetic agent was determined based on the GC–MS analysis, focusing on whether the components in the formulation would produce toxic effects in fish. The anesthetic mixture is registered by NatiVital company (YK-TR-3401052).

**Table 1:** Component of commercial anesthetic agent.

Peak	Constituents	Retention time	Area (%)	Height (%)
1	2-Ethoxy-1-propanol	3.8580	17.4	12.4
2	5-Methyl-3-heptanone	10.541	0.29	0.46
3	(Z)-β-Ocimene	12.075	0.28	0.40
4	1,8-Cineole	12.151	0.54	0.89
5	(E)-β-Ocimene	12.511	1.60	2.56
6	3,7-Dimethyl-1,6-octadien-3-ol	14.941	9.22	12.8
7	3-Acetoxy-1-octene	15.382	0.30	0.47
8	4-(p-Mentha-1,4-dien-1-yl)-3-ol	17.814	1.13	1.57
9	α-Terpineol	18.347	0.76	0.93
10	3,7-Dimethyl-1,6-octadien-3-yl acetate	20.750	9.30	13.3
11	2-Isopropenyl-5-methylhex-4-en-1-yl acetate	22.003	1.97	2.90
12	4-(tert-Butyl)phenol	22.549	1.71	2.10
13	4-Allyl-2-methoxyphenol	24.434	40.8	31.3
14	(Z)-3,7-Dimethyl-2,6-octadien-1-yl acetate	24.565	0.31	0.41
15	(E)-3,7-Dimethyl-2,6-octadien-1-yl acetate	25.216	0.36	0.52
16	1-Methyl-1-vinyl-2,4-diisopropylcyclohexane	26.453	5.40	7.08
17	(E)-β-Farnesene	27.645	1.80	1.99
18	4-Allyl-2-methoxyphenyl acetate	29.910	6.22	7.17
19	(−)-Caryophyllene oxide	31.683	0.46	0.62
			100	100

### 2.2 Experimental design

The current study was approved by Ethical Committee of Recep Tayyip Erdoğan University, Türkiye (Decision No: 2020/37). All experimental studies were carried out at Recep Tayyip Erdoğan University Aquaculture Research and Application Center. In the current study, fish belonging to three different trophic levels were used: juvenile individuals of rainbow trout (85.4 ± 6.1 g), common carp (49.5 ± 4.7 g), and Danube sturgeon (55.8 ± 6.2 g). The fish were adapted to the experimental environment 1 week before trial. Groundwater with a constant water temperature of 18°C was used. For these fish species belonging to different trophic levels, water temperatures normally vary. However, a water temperature that could provide a common adaptation for all fish species was selected. Fish were exposed to three different concentrations of anesthetic agents: low (LC), medium (MC) and high (HC) concentration. LC, MC, and HC concentration were 300, 400, and 500 mg L^−1^ for rainbow trout; 240, 360, and 480 mg L^−1^ for common carp; and 600, 675, and 750 mg L^−1^ for Danube sturgeon, respectively. A preliminary study was conducted to determine the concentrations, and the concentrations that produced sedative, anesthetic, and deep anesthetic effects were identified beforehand. The fish were fasted for 36 h before the trial and were not fed during the study. Anesthesia treatment was carried out triplicate with 10 fish individually in 50 L tanks (cylinder and fiberglass). Reactions such as total loss of reaction to the stimulus, irregular opercular movements, and loss of balance were considered to determine the induction time (21). Recovery represents the moment of active balanced swimming and response to stimulation (22). Anesthesia concentrations were determined to ensure anesthesia within a maximum of 5 min. For recovery after induction, anesthetized fish were quickly placed in 500 L tanks that were strongly aerated with an air stone (DO~10 mg L^−1^). In order to ensure standardization, induction and recovery times were recorded by a single researcher.

### 2.3 Hematological examination

Blood was sampled based on time and concentration variables (2 × 4 matrix) for hematological studies. Namely, samples were taken at 0th, 2nd, 4th, and 8th h after recovery for all concentration groups. Fish (six individuals) were randomly selected and blood samples were taken from the caudal vein with a 2.5 mL syringe. Blood was transferred to EDTA K3 tubes to prevent clotting and to preserve it for hematological studies. Erythrocyte (RBC), leukocyte (WBC), hematocrit (HCT), and hemoglobin (HGB) concentrations were measured with an automatic hematological assay (Prokan6800VET). The instrument was calibrated with blank blood samples of the relevant healthy fish before the study (23).

### 2.4 Histological assessment

Histological examination was evaluated in the gill tissues of anesthetized fish. Anesthetized fish were euthanized with mechanical stunning method and tissues were later sampled. Control fish were euthanized after mechanical stunning. Three individual from each group of each fish species were histologically examined. Tissues were fixed in 10% neutral buffer formalin. After 48 h, fixed tissues were preserved in 50% ethyl alcohol. Gill tissues were treated in alcohol series and stored overnight in liquid paraffin at 65°C. Afterwards, the samples were embedded in paraffin. Samples with a thickness of 5 microns were taken to microscope slide from paraffin-blocked tissues using a microtome. The preparations, whose paraffin was removed at 65°C, were subjected to alcohol and xylene series. Then, tissue sections were stained with hematoxylin and eosin. Stained tissues were fixed with Entellan^®^ (MERCK/107961.0500) and covered by cover-slip. Histopathologic changes were examined with light microscope (24). Changes in fish gill tissues due to exposure to commercial anesthetic agent were determined by comparing with control group gill tissues.

### 2.5 Multi-criteria decision-making model

Multi-criteria decision-making models (MCDM) were used to determine which concentration was preferable for the commercial anesthetic mixture. This model consists of four basic steps (25): (1) investigation of the anesthetic mixture for three fish species, (2) determination of evaluation criteria and weights for each concentration group, (3) scoring the anesthetic concentrations based on evaluation criteria, and (4) Decision the best anesthetic concentration via PROMETHEE decision model. According to this model, a set of evaluation criteria were considered for each alternative. Each evaluation criteria consists of weight value and sum of the weight values is “1” (It means percentage importance of criteria).

The PROMETHEE method was selected in this study due to its suitability for comparing alternatives that involve both qualitative and quantitative criteria. Specifically, the method allows for the integration of qualitative data such as histological findings and quantitative data like blood parameters, which are crucial for a comprehensive evaluation. PROMETHEE’s robust comparison mechanism not only provides insights into the strengths and weaknesses of each alternative but also enhances decision-making by highlighting the relative importance of each criterion. Moreover, the method is supported by software that can visualize the rankings, making the results more transparent and interpretable. Given these advantages, PROMETHEE is the most appropriate approach for analyzing the multi-criteria data in this study (25, 26).

#### 2.5.1 Determination of evaluation criteria and its weight percentage

Based on MCDM principle, alternatives must evaluate according to determined criteria. Because the decision maker makes an evaluation and reaches a conclusion by taking these criteria into account. Evaluation criteria for determining the best anesthetic concentration are considered based on feasibility, cost, effectiveness of the anesthetic agent and physiological state of the fish. In the current study, 10 different evaluation criteria were determined. Evaluation criteria were determined under four main headings: (1) induction and recovery times, (2) cost analysis, (3) hematological parameters, and (4) histological alterations. In the model, quantitative and only those qualitative parameters that could be quantified were used in determining the evaluation criteria. Depending on the importance factor of each criterion, the weight value was determined by an expert opinion. The weights of the criteria were determined through a structured and transparent process to ensure accuracy in the MCDM analysis. A panel of experts, each with extensive knowledge in aquaculture and fish welfare, was consulted to assign relative importance to each criterion. These experts were selected based on their years of experience, professional background, and expertise in areas related to both histological and blood parameters. To aggregate the opinions and assign the weights, we employed the Analytic Hierarchy Process (AHP). This method was chosen because it systematically captures expert judgment and minimizes biases in the weighting process. A total of 15 different experts independently evaluated the criteria. The analysis of the anesthetic effects of a commercial agent across three different fish species reveals a Consistency Index (CI) of 0.017 and a Consistency Ratio (CR) of 0.011, indicating an acceptable level of consistency in the prioritization of concentrations based on various criteria. These findings underscore the robustness of the concentration rankings established for the evaluation of the anesthetic agent’s efficacy. The evaluation criteria and their weights are presented in Table 2. In PROMETHEE software, the weight scores of the criteria must be 1 (27). Therefore, weight scores are distributed according to the importance of the criteria. The most effective criteria in choosing the best anesthetic concentration are induction time, cost and necrosis. Because the induction time of the fish is the primary parameter showing the effectiveness of the anesthetic agent, cost is the indicator of feasibility, and necrosis is the most important histological finding. Recovery time has a lower weight value, because the induction time and recovery time should be <3 and < 5 min, respectively (28). Since WBC is an indicator of the fish’s defense mechanism and RBC is a representative of the amount of oxygen in the fish’s blood, it has a significance value of 10%.

**Table 2:** Evaluation criteria and weighting scale.

Criteria number	Evaluation criteria	Weight value	Preference function
C1	Induction time	0.15	Linear
C2	Recovery time	0.10	Linear
C3	Cost	0.15	V-shape
C4	WBC	0.10	Linear
C5	RBC	0.10	V-shape
C6	HGB	0.05	V-shape
C7	HCT	0.05	Linear
C8	Necrosis	0.15	Usual
C9	Hyperplasia	0.075	Level
C10	Hypertrophy	0.075	Level

Continuously variable seconds were considered as induction and recovery time. For cost analysis, 1 L of anesthetic agent was accepted as 120 USD ($) and normalized according to the amount of anesthetic agent used for each fish species. The average value of the relevant group at the eighth hour was evaluated for blood parameters. For histological alterations, a five-point Likert scale indicating the severity of the lesion was used. Decision matrices for three fish species, considering criteria and alternatives, are provided in Table 3.

**Table 3:** Decision matrix based on criteria and alternatives (C1-C10 represents criteria numbers).

		C1	C2	C3	C4	C5	C6	C7	C8	C9	C10
Rainbow trout	LC	206.00	99.60	1.80	26.60	1.33	11.93	22.56	1/5	1/5	1/5
	MC	173.00	130.60	2.40	21.70	1.17	10.10	17.90	3/5	3/5	1/5
	HC	110.90	201.70	3.00	23.13	1.32	10.97	20.03	4/5	3/5	1/5
Common carp	LC	247.30	94.30	1.44	10.83	1.84	9.53	27.66	1/5	1/5	1/5
	MC	198.00	113.90	2.16	11.93	2.05	10.50	28.06	1/5	3/5	1/5
	HC	139.10	162.90	2.88	10.90	1.63	9.53	25.46	1/5	4/5	1/5
Danube sturgeon	LC	226.50	98.00	3.60	39.50	0.79	9.36	15.80	1/5	1/5	3/5
	MC	200.90	124.70	4.04	37.96	0.77	9.20	15.80	3/5	3/5	1/5
	HC	154.90	213.50	4.50	39.33	0.78	8.73	15.63	5/5	4/5	1/5

#### 2.5.2 Selection and ranking of best alternatives by PROMETHEE analysis

The best concentration of a commercial anesthetic agent for three different fish species was determined with the PROMETHEE decision model (Visual PROMETHEE 1.1.0.0). After the evaluation criteria and the weight values of these criteria were determined, a decision matrix was created. PROMETHEE allows decision makers to make a specific choice in terms of an evaluation factor or to limit the evaluation factor to values they specify. The preference functions were selected based on the type of evaluation criteria (quantitative, qualitative, or Likert) and their sensitivity. The preference threshold was determined to reflect a significant difference within the context of our research. This threshold was established through discussions with subject matter experts, who provided insights into what constitutes a meaningful improvement in the evaluated criteria. The indifference threshold was defined based on expert consultations and practical evaluations. We aimed to identify a range where the differences between alternatives are negligible and do not influence the decision-making process. This threshold helps to indicate that the alternatives can be considered equivalent, thereby simplifying the decision-making process. PROMETHEE decision model is a method that reaches results in a total of seven steps.

**Step one:** The data matrix is prepared using Equations 1–3. Criterion weights are determined for *k* number criteria (*k* = 10 in the current study).


(1) $$w=w_{1},w_{2},\ldots ,w_{k}$$

w:criteriaweight

(2) $$c=f_{1},f_{2},\ldots ,f_{k}$$

c:criteriaweight and f:function


(3) $$S=\left(A,B,C,\ldots \right)$$

S:decisionalternatives

**Step two:** Preference functions for the criteria are determined according to Equation 4 (linear preference function) and Equation 5 (usual preference function).


(4) $$p\left(d\right)=\{\begin{array}{cc} 0 & d\leq q\\ \left(d-p\right)/\left(p-q\right) & q<d\leq p\\ 1 & d>p \end{array}\left(d-p\right)/\left(p-q\right)$$

q:indifferencevalue

p:sufficientbiggestdifference

q:differencebetweentwodecisionalternatives

(5) $$p\left(d\right)=\{\begin{array}{cc} 0 & d\leq 0\\ 1 & d>0 \end{array}$$

**Step three:** The common preference function for decision alternatives “x” and “y” is calculated with Equation 6.


(6) $$p\left(x,y\right)=\{\begin{array}{cc} 0 & f\left(x\right)\leq f\left(y\right)\\ p\left[f\left(x\right)-f\left(y\right)\right] & f\left(x\right)>f\left(y\right) \end{array}$$

According to Equation 6, it is determined whether the evaluation factor is maximization or minimization.

**Step four:** The preference index of “x” and “y” decision options evaluated according to the k-number criterion was calculated using Equation 7.


(7) $$\pi \left(x,y\right)=\overset{K}{\underset{i=1}{\sum }}w_{i}P_{i}\left(x,y\right)$$

**Step five:** Determining positive *φ*^+^ and negative φ^−^ advantages for alternatives with Equations 8 and 9.


(8) $$\varphi^{+}\left(x\right)=\frac{1}{n-1}\sum \pi \left(x,y\right)$$

(9) $$\varphi^{-}\left(x\right)=\frac{1}{n-1}\sum \pi \left(y,x\right)$$

**Step six:** Partial priorities are determined with PROMETHEE I. Equations 10 and 11 show the difference between “x” and “y” decision alternatives. If any of the following conditions occur, decision option “x” is indistinguishable from decision option “y.”


(10) $$\varphi^{+}\left(x\right)=\varphi^{+}\left(y\right)$$

(11) $$\varphi^{-}\left(x\right)=\varphi^{-}\left(y\right)$$

Similar to the example below, if any of the conditions in Equations 12–14 occur, the “x” decision option is superior to the “y” decision option.


(12) $$\varphi^{+}\left(x\right)>\varphi^{+}\left(y\right)\;\mathrm{and}\;\varphi^{-}\left(x\right)<\varphi^{-}\left(y\right)$$

(13) $$\varphi^{+}\left(x\right)>\varphi^{+}\left(y\right)\;\mathrm{and}\;\varphi^{-}\left(x\right)=\varphi^{-}\left(y\right)$$

(14) $$\varphi^{-}\left(x\right)<\varphi^{-}\left(y\right)\;\mathrm{and}\varphi^{+}\left(x\right)=\varphi^{+}\left(y\right)$$

In the condition where decision alternative “x” cannot be compared with decision alternative “y,” Equations 15 and 16 are used.


(15) $$\varphi^{+}\left(x\right)>\varphi^{+}\left(y\right)\;\mathrm{and}\;\varphi^{-}\left(x\right)>\varphi^{-}\left(y\right)$$

(16) $$\varphi^{+}\left(x\right)<\varphi^{+}\left(y\right)\;\mathrm{and}\;\varphi^{-}\left(x\right)<\varphi^{-}\left(y\right)$$

**Step seven:** The ranking of decision options is performed with PROMETHEE II. The exact priorities of the decision options are determined by Equation 17. All calculated priority values are sorted from high priority to low priority. Thus, all decision options are evaluated in a similar way and a complete ranking is obtained.


(17) $$\varphi \left(x\right)=\varphi^{+}\left(x\right)-\varphi^{-}\left(x\right)$$

The decisions given in Equations 18 and 19 can be reached according to the full priority value calculated from the “x” and “y” decision alternatives.


(18) $$\varphi \left(x\right)>\varphi \left(y\right)$$

Decision alternative “x” is superior.


(19) $$\varphi \left(x\right)=\varphi \left(y\right)$$

Decision alternatives “x” and “y” are not superior.

### 2.6 Statistical analysis

All data are presented as the means ± standard deviation (SD). For hematological studies, two-way multivariate analysis of variance (MANOVA) test was applied based on both concentration and time variables. Levene test was used to determine the equality of variances, and Tukey test was considered to determine the difference between groups. Differences were considered statistically significant when the calculated *p* value was <0.05. All analyses were performed in SPSS software (Version 23, IBM Corp., Armonk, New York, United States).

## 3 Results

### 3.1 Induction and recovery times

**Figure 1:**
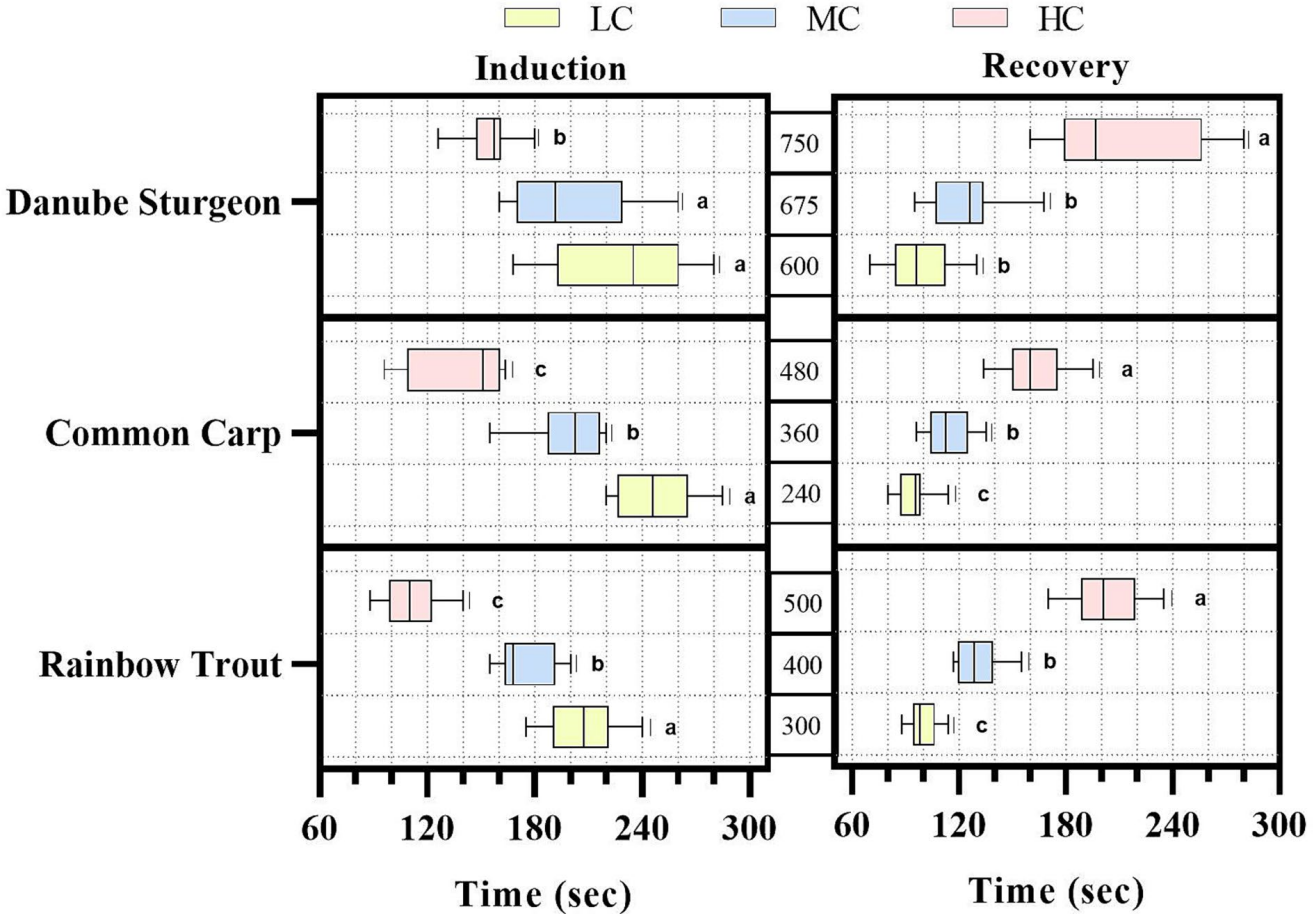
Induction and recovery times of three different fish species against anesthetic agents. ^abc^Indicates a statistically significant difference.

Induction and recovery times for three fish species are shown in Figure 1. Accordingly, concentrations for both induction and recovery were determined to be between 1 and 5 min. The fastest response to commercial anesthetics was rainbow trout, followed by Danube sturgeon and common carp, respectively. According to recovery times, Danube sturgeon takes the longest time, especially for high concentration, followed by common carp and rainbow trout, respectively. Induction and recovery times differed significantly for rainbow trout and common carp according to concentration (*p* < 0.01). In Danube sturgeon, high concentration anesthetic mixture showed significantly faster induction and slower recovery time than MC and LC (*p* < 0.01).

### 3.2 Histological findings

**Figure 2:**
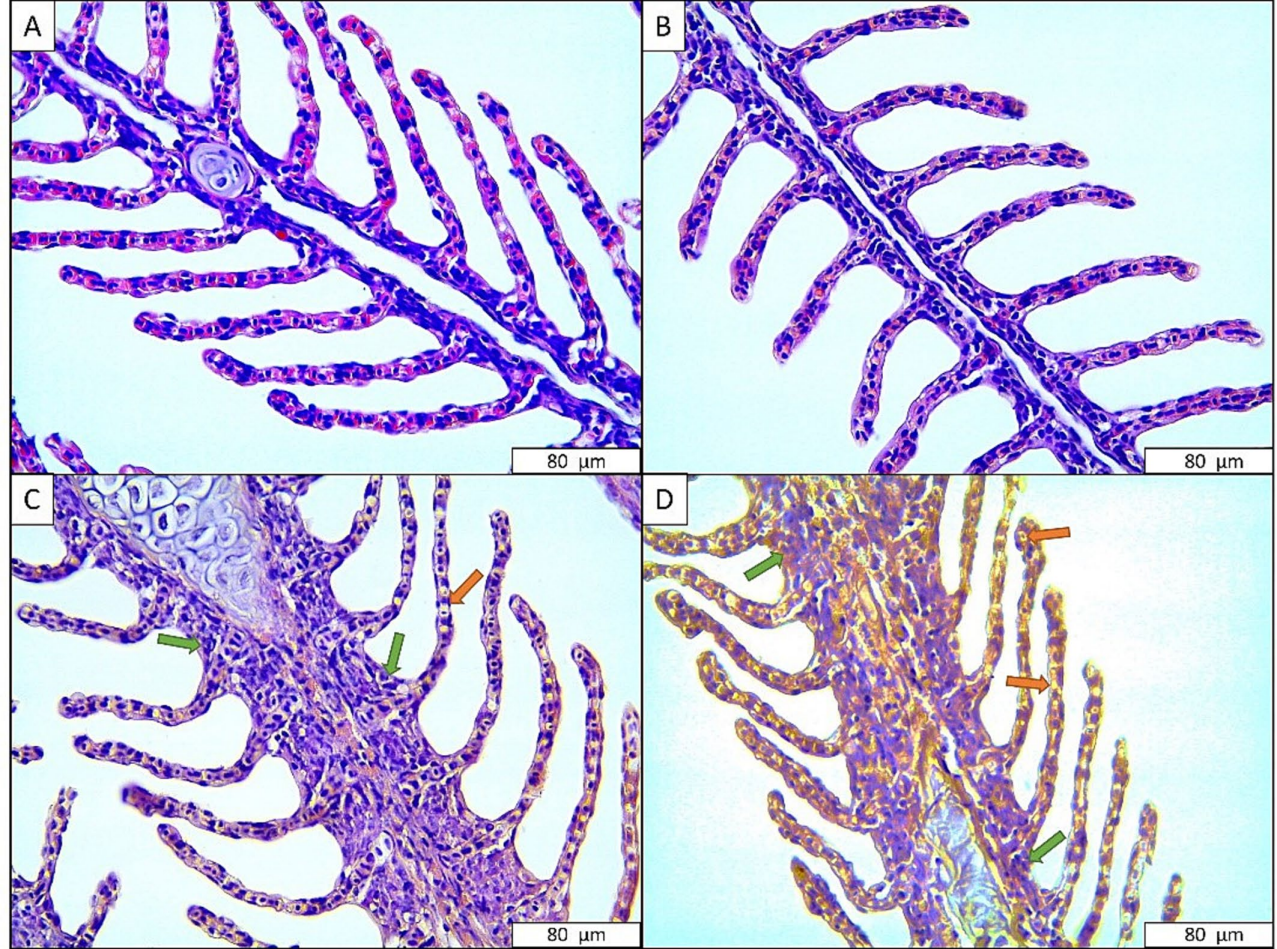
Gill tissues of rainbow trout exposed to anesthetic agent. (A) Control; (B) 300 mg L^−1^ anesthetic agent; (C) 400 mg L^−1^ anesthetic agent; (D) 500 mg L^−1^ anesthetic agent. Green arrow: hyperplasia, Orange arrow: necrosis.

The gill tissues of rainbow trout exposed to the anesthetic mixture are shown in Figure 2. Accordingly, no abnormal signs were observed in the gills of the fish in the control group and the fish exposed to 300 mg L^−1^ anesthetic mixture. On the other hand, signs such as necrosis and hyperplasia were observed in the gill tissues of fish exposed to 400 mg L^−1^ (moderate) and 500 mg L^−1^ (severe) anesthetic mixture (Table 4).

**Table 4:** Severity of different histological changes in gill tissues.

		Control	LC	MC	HC
Rainbow trout	Hyperplasia	−	−	++	+++
	Necrosis	−	−	++	+++
Common carp	Hyperplasia	−	−	++	++++
Danube sturgeon	Hyperplasia	+	++	++	++
	Necrosis	−	−	++	++++
	Hypertrophy	−	++	+	+

(−), none; (+), mild; (++), moderate; (+++), severe; (++++), very severe.

**Figure 3:**
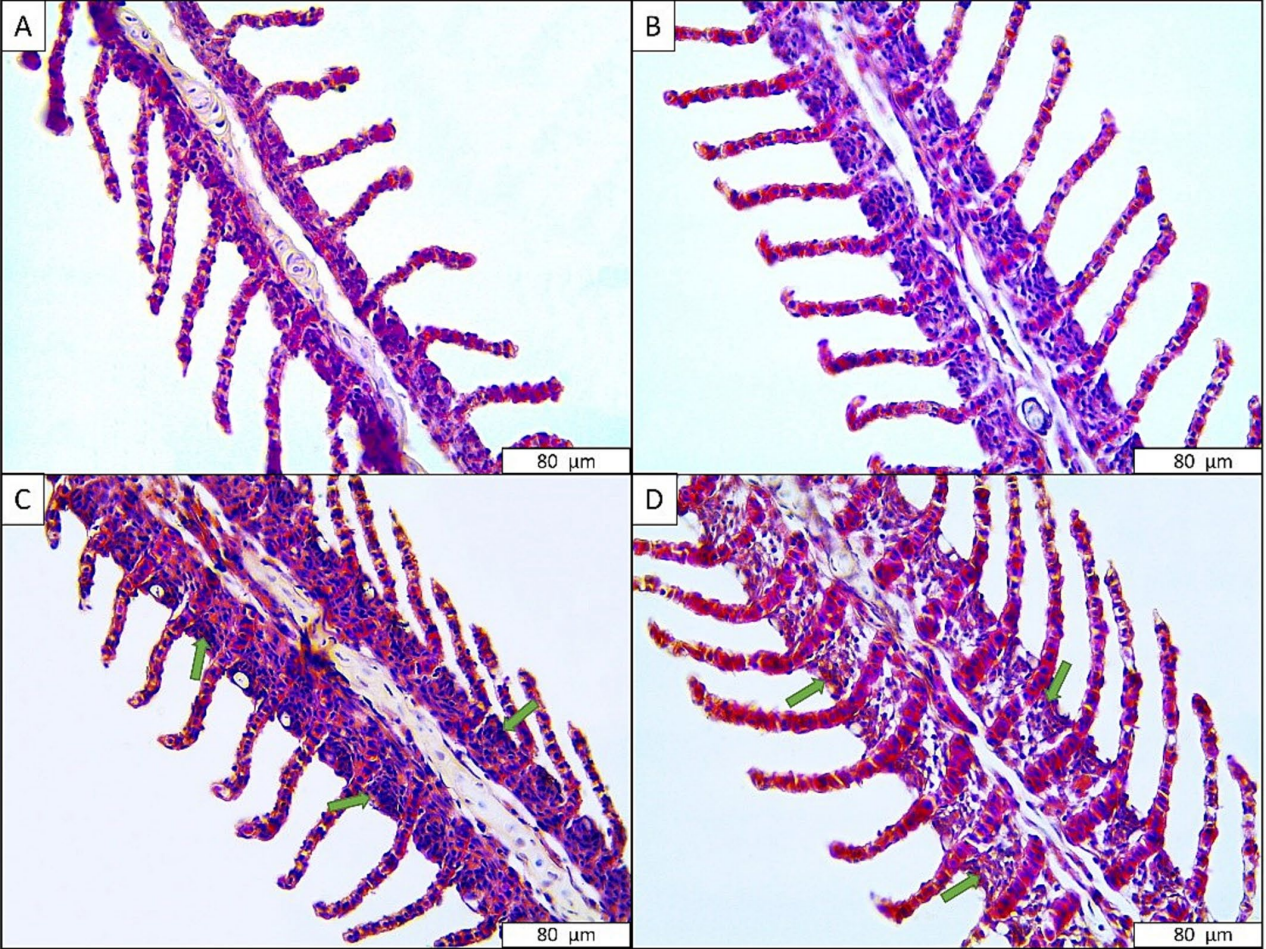
Gill tissues of common carp exposed to anesthetic agent. (A) Control; (B) 240 mg L^−1^ anesthetic agent; (C) 360 mg L^−1^ anesthetic agent; (D) 480 mg L^−1^ anesthetic agent. Green arrow: hyperplasia.

No histological change was observed in the common carp gill tissues in the control group and low anesthetic concentration group, similar to rainbow trout (Figure 3). However, sings of hyperplasia, especially in the primary lamellae, were observed in fish exposed to 360 mg L^−1^ (moderate) and 480 mg L^−1^ (very severe) anesthetic mixtures (Table 4).

**Figure 4:**
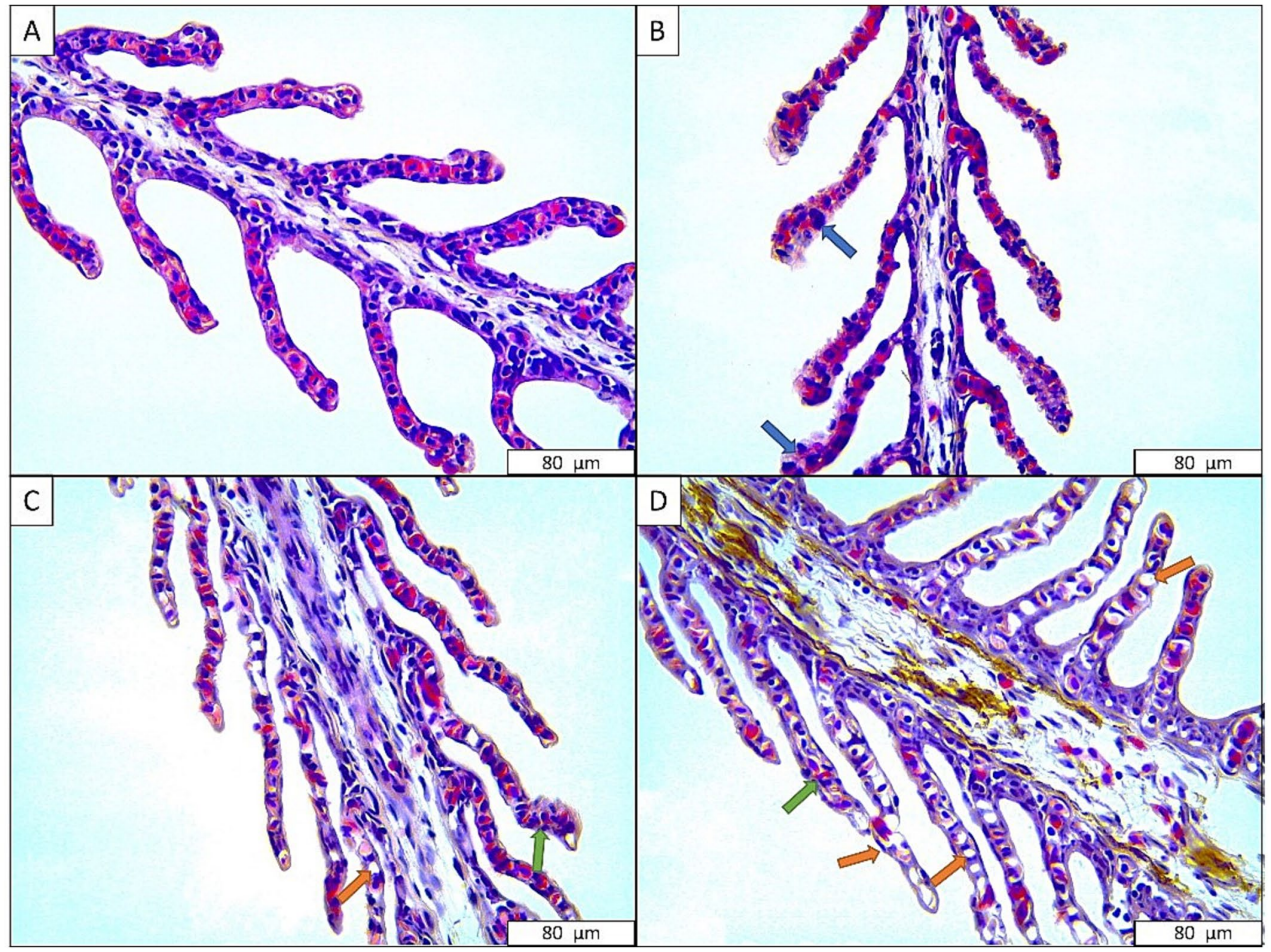
Gill tissues of Danube sturgeon exposed to anesthetic agent. (A) Control; (B) 600 mg L^−1^ anesthetic agent; (C) 675 mg L^−1^ anesthetic agent; (D) 750 mg L^−1^ anesthetic agent. Blue arrow: hypertrophy, Green arrow: hyperplasia, Orange arrow: necrosis.

Figure 4 presents histological images of the gills of sturgeon exposed to the anesthetic agent. While there is no histological alteration in the control group, hypertrophic cells were observed in the group exposed to 600 mg L^−1^ anesthetic mixture. Additionally, hyperplasia and severe necrosis sings were also recorded in groups exposed to 675 and 750 mg L^−1^ anesthetic (Table 4).

### 3.3 Hematological evolution

WBC, RBC, HGB, and HCT values were evaluated as hematological parameters for three fish species (Table 5). Blood parameters were taken from fish in LC, MC, and HC groups at the 0, 2, 4, and 8 h after anesthesia. Accordingly, significant differences were observed in WBC and HCT in rainbow trout only over time (*p* < 0.01). This difference is due to significantly higher levels of WBC and HCT at hour 0 for the rainbow trout. In common carp, significant differences were observed for WBC and HCT depending on time, and for WBC, RBC, and HGB according to concentration (*p* < 0.01). In common carp, higher concentrations have shown significantly higher WBC, RBC, and HGB levels. Over time, the longer duration after anesthesia has increased WBC and HCT while decreasing HGB. In Danube sturgeon, while significant differences were observed over time for the whole blood parameter, this difference was not observed for concentration. For Danube sturgeon, the time variable exhibited a similar scenario for all blood parameters, initially decreasing and then rising back to the same levels over time.

**Table 5:** Statistical evaluation of hematological parameters based on concentration and time.

	Rainbow trout			Common carp			Danube sturgeon		
	LC	MC	HC	LC	MC	HC	LC	MC	HC
WBC (10^3^ μL^−1^)									
0^th^ h	27.6 ± 3.2	29.5 ± 0.9^a^	30.4 ± 2.4	9.3 ± 1.8	7.0 ± 1.0^c^	9.0 ± 1.4^ab^	35.9 ± 1.6	41.4 ± 2.4	36.9 ± 3.1^a^
2^nd^ h	26.5 ± 3.0	28.5 ± 2.8^ab^	29.5 ± 4.4	10.6 ± 2.5^AB^	10.8 ± 0.3^Ab^	7.0 ± 0.8^Bb^	35.8 ± 3.1	34.6 ± 4.3	29.5 ± 2.6^b^
4^th^ h	25.1 ± 1.2^AB^	28.6 ± 3.0^Aab^	21.7 ± 1.7^B^	10.0 ± 1.2^B^	13.7 ± 0.5^Aa^	10.3 ± 1.1^Ba^	37.9 ± 6.3	36.9 ± 3.8	37.9 ± 1.7^a^
8^th^ h	26.6 ± 5.6	21.8 ± 3.4^b^	23.1 ± 5.2	10.8 ± 1.3	11.9 ± 1.2^ab^	10.9 ± 0.3^a^	39.5 ± 4.1	38.0 ± 2.6	39.3 ± 0.9^a^
RBC (10^6^ μL^−1^)									
0^th^ h	1.4 ± 0.2	1.4 ± 0.1	1.4 ± 0.1	2.0 ± 0.2	1.7 ± 0.1^b^	1.9 ± 0.1	0.7 ± 0.1	0.8 ± 0.1	0.7 ± 0.1
2^nd^ h	1.3 ± 0.1	1.5 ± 0.2	1.4 ± 0.1	1.7 ± 0.4	1.9 ± 0.1^ab^	1.6 ± 0.3	0.7 ± 0.1	0.7 ± 0.1	0.5 ± 0.2
4^th^ h	1.3 ± 0.1	1.4 ± 0.2	1.2 ± 0.1	1.8 ± 0.1^B^	2.2 ± 0.1^Aa^	1.7 ± 0.1^B^	0.7 ± 0.1	0.8 ± 0.1	0.8 ± 0.1
8^th^ h	1.3 ± 0.2	1.2 ± 0.1	1.3 ± 0.2	1.8 ± 0.1^AB^	2.1 ± 0.1^Aa^	1.6 ± 0.1^B^	0.8 ± 0.1	0.8 ± 0.1	0.8 ± 0.1
HGB (g dL^−1^)									
0^th^ h	11.7 ± 1.0	12.1 ± 0.4	12.0 ± 0.3	10.8 ± 0.2^AB^	10.2 ± 0.3^B^	11.1 ± 0.3^Aa^	8.7 ± 0.8	10.2 ± 1.0	8.9 ± 0.6^ab^
2^nd^ h	10.9 ± 0.6	12.3 ± 1.2	12.7 ± 1.3	10.1 ± 1.3^AB^	11.4 ± 0.4^Aa^	8.4 ± 1.1^Bb^	8.5 ± 0.8	8.2 ± 1.2	5.8 ± 2.1^b^
4^th^ h	11.1 ± 0.9	12.2 ± 0.8	13.4 ± 4.3	10.1 ± 0.5	10.9 ± 0.5^ab^	9.7 ± 0.6^ab^	8.6 ± 1.8	8.6 ± 0.9	9.1 ± 0.4^a^
8^th^ h	11.9 ± 1.7	10.1 ± 1.1	11.0 ± 1.7	9.6 ± 0.5	10.5 ± 0.5^ab^	9.6 ± 0.7^ab^	9.6 ± 0.6	9.2 ± 0.5	8.7 ± 0.4^a^
HCT (%)									
0^th^ h	23.0 ± 2.6	25.5 ± 1.1^a^	24.2 ± 0.3^ab^	25.4 ± 3.1	23.2 ± 2.2^c^	24.8 ± 2.4	14.4 ± 0.6	16.9 ± 1.3	14.6 ± 1.9
2^nd^ h	21.1 ± 1.8	22.5 ± 2.1^a^	24.6 ± 1.0^a^	23.1 ± 4.6	24.3 ± 0.7^bc^	23.7 ± 1.8	14.4 ± 1.2	14.1 ± 2.4	9.9 ± 4.9
4^th^ h	19.7 ± 2.5	21.7 ± 1.0^ab^	19.2 ± 0.5^b^	29.9 ± 3.1	29.2 ± 1.7^a^	26.5 ± 2.5	15.0 ± 3.0	15.2 ± 1.8	15.4 ± 0.4
8^th^ h	22.6 ± 5.1	17.9 ± 2.0^b^	20.0 ± 3.8^ab^	27.7 ± 1.6	28.1 ± 1.5^ab^	25.5 ± 1.3	15.8 ± 2.4	15.8 ± 1.5	15.6 ± 0.3

Capital letters present significant differences between concentration groups according to time. Lower cases present significant differences between time groups according to concentration.

### 3.4 PROMETHEE decision model

**Figure 5:**
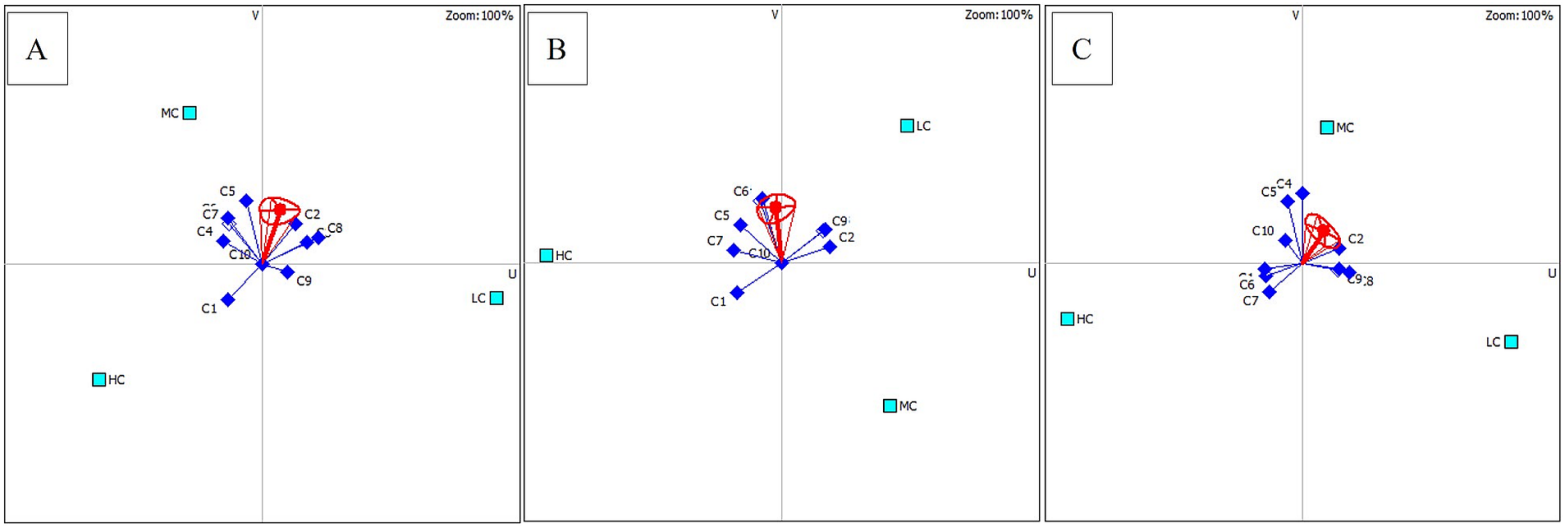
Distribution of criteria for three different fish species according to the PROMETHEE decision model. (A) Rainbow trout; (B) Common carp; (C) Danube sturgeon.

The best option of anesthetic concentrations used for three different fish species was determined by the PROMETHEE decision model (Table 6). According to the rankings made considering *φ(i)*, the most suitable anesthetic agent concentrations for rainbow trout, common carp, and Danube sturgeon are MC, LC, and MC, respectively. Additionally, the higher concentration was the least preferable concentration for all groups. The distribution of criteria affecting concentration ranking is shown in Figure 5. The main parameters that make MC concentration a priority for rainbow trout are blood parameters and relatively cost. For common carp, LC is ranked first due to shorter recovery time, lower cost and mild hyperplasia. Finally, the priority of MC for Danube sturgeon is related to WBC, RBC and recovery time.

**Table 6:** Ranking of concentration groups for three different fish species.

Fish	Concentration group	*φ*^+^(*i*)	*φ*^−^(*i*)	*φ*(*i*)	Ranks
Rainbow trout	MC	0.3696	0.1844	0.1853	1
	LC	0.3590	0.2904	0.0686	2
	HC	0.1806	0.4344	−0.2538	3
Common carp	LC	0.3400	0.1611	0.1789	1
	MC	0.3076	0.2634	0.0442	2
	HC	0.1361	0.3592	−0.2231	3
Danube sturgeon	MC	0.3933	0.2398	0.1353	1
	LC	0.3837	0.3225	0.0612	2
	HC	0.2649	0.4796	−0.2147	3

## 4 Discussion

The ideal anesthetic agent should not be physiologically toxic to the fish and should not adversely affect human health during operating, and should have an appropriate induction and recovery time for the fish (29). Because long-term or high-concentration exposure to the anesthetic substance is important both in terms of operating cost and stress for the fish (30). In the current study, studies were first conducted with 80–100 mg L^−1^, which was determined as the suitable concentration of the anesthetic mixture. However, the determined concentrations did not provide effective application in fish. Although the concentration was increased, the anesthetic concentration of 250 mg L^−1^ for carp and 240 mg L^−1^ for rainbow trout did not produce the expected induction effect. In accordance with the preliminary studies conducted for Danube sturgeon, a mild sedation effect was not observed within 5 min for 240, 360, and 480 mg L^−1^. Therefore, the lowest concentration for Danube sturgeon has been increased to 600 mg L^−1^. The expected anesthetic effect in fish was achieved only at this concentration. This issue reveals Danube Sturgeon’s resistance to the anesthetic mixture. As a result, HC concentrations were induced for all fish in less than 3 min. MC concentrations generally represent the minimum possible concentration for anesthesia. HC concentrations for common carp, rainbow trout, and Danube sturgeon were determined as 480, 500, and 750 mg L^−1^, respectively. Common carp and rainbow trout showed similar anesthesia behavior at similar concentrations. However, although similar concentrations were used, the induction time was faster in rainbow trout than in common carp. Similarly, a slower induction time has been reported for common carp than rainbow trout (14, 31, 32). On the other hand, even the lowest anesthesia concentration of Danube sturgeon was recorded higher than the highest concentration of other groups. Although the anesthetic effect of chamomile oil was determined for rainbow trout in our previous study, similar concentrations did not show an anesthetic effect for Danube sturgeon (11). Wide range in anesthetic concentration is associated with fish species, size and environmental conditions (33–35). Therefore, we focused on the effect of fish species on anesthetic concentration determination, keeping fish sizes and environmental conditions constant. Smaller fish have a faster induction rate, while larger fish show a faster recovery time (4). Thus, combination anesthesia may be safer because this allows a reduction in dose, which is generally reflected in better recovery and lower mortality rates along with reduced adverse side effects in some cases (36). The anesthetic mixture should have a pleasant smell for the users (29). The commercial anesthetic mixture used in the current study has an aromatic odor and is not irritating to users. Its smell has an intense scent of thyme and clove. However, the current anesthetic mixture can be added directly to water due to unlike essential oils, it has the ability to dissolve in water. Therefore, it does not create a synergistic effect on fish like water-insoluble essential oils. The anesthetic agent suitable for fish should not cause death even after a 30-min induction period. According to the reliability test applied in this study, no mortality was observed in fish for optimum concentrations. As expected, recovery times increased significantly in all fish due to increasing concentration.

Anesthetics first come into contact with the gills in fish and are distributed throughout the body via this route. Therefore, histopathological studies are critical to confirm sensitivity to the gill epithelium, which is the predominant site of gas exchange and osmoregulation (37). In the present study, rainbow trout and common carp gill tissues were little affected by low anesthetic concentration. However, the commercial anesthetic mixture had a histological effect on Danube sturgeon gills even at low concentrations. Hypertrophic cells were observed moderately in the gill tissues of Danube sturgeon. In the MC groups, while rainbow trout and common carp gill tissues showed similar signs of moderate hyperplasia and necrosis, severe hyperplasia was observed in Danube sturgeon. For HC groups, severe and/or very severe hyperplasia and necrosis were observed in the gill tissues of all fish species. In general, the more severe histological output of Danube sturgeon than other species has been attributed to the higher anesthetic agent concentration. The contribution of increasing anesthetic agent concentration to histological degradation has been discussed previously (11). Hypertrophy of the secondary lamella and hyperplasia of the primary lamella in the current study are a common and nonspecific response to subacute and chronic damage to the gills (38, 39). These cell responses are among the first defense mechanisms and are classified as mild and reparable (40). The potential hyperplasia and hypertrophy effects of different herbal anesthetic essential oils, such as the content of the commercial anesthetic substance in our study, on gill tissues have been reported (26, 41).

Exposure to anesthesia slows down operculum movement in fish and causes hypoxia. The increase in hypoxia affects catecholamine in rainbow trout, resulting in greater RBC release from the spleen as well as an increase in hematocrit and hemoglobin (42). Similarly, higher RBC, HGB, and HCT were observed in the anesthesia groups at the beginning of experiment than in the control, and blood parameters increased further depending on increasing anesthesia concentration. Blood parameters returned to control-like levels over time. However, while significant differences were observed over time in the MANOVA test, fewer differences were observed depending on concentrations. It has been inferred that that this increase in blood parameters negatively affects fish welfare in the acute period after anesthesia and that the first 24 h in anesthetized individuals are critical. In one study, the lymphocyte ratio reached initial levels within 24 h after anesthesia, supporting our hypothesis (43). It has been previously discussed that clove oil, an herbal anesthetic agent, increases RBC (44). It may cause malfunctions in the hematopoietic system of fish exposed to the potentially toxic substance, resulting in increased WBC and HGB (45). The interaction effect showed significant differences between groups depending on one and/or two variables only for WBC, RBC, and HGB in carp. For instance, although there was no significant difference for the time variable, significant differences in concentration put pressure on the time variable.

PROMETHEE decision model is widely used in many fields due to its mathematical properties and easy applicability. Because it is a very well designed model for ranking a limited number of alternatives according to conflicting criteria (46). Although it has been applied in many areas, the PROMETHEE decision model is a design that has not yet been applied in the field of aquaculture. In the current study, three anesthesia concentration alternatives were evaluated for three different fish species based on 10 criteria. While MC showed the best results for rainbow trout and Danube sturgeon, LC group provided the top rank for common carp. Here, the weight values of the criteria have an important share and these values are determined according to expert opinion. C1 and C2 are the criteria that reveal the effectiveness of the anesthetic agent, and the weight value is determined as 15 and 10% for induction and recovery time, respectively. Because while the induction time is expected to be <3 min, the recovery time has a wider range of <5 min (28). C3 represents a part of the operating cost, which is one of the most important items for the employer in the aquaculture industry. As the running cost, the amount of anesthetic mixture is a significant input cost in full-scale facilities, depending on the volatility of the essential substance, the size of the water volume and the amount of fish. From a public policy perspective, potential positive or negative impacts should be considered on a cost–benefit basis (47). As physiological indicators of fish, criteria between C4 and C7 represent blood parameters that can provide rapid results (48). The weight values of WBC, which is a representative of the defense mechanism of the fish, and RBC, which transports oxygen in the blood, were determined higher. Finally, C8, C9, and C10 are criteria that indicate histological changes. The reason for choosing a higher weight value for necrosis (C8) is that it represents a significant and irreversible histological change in which the cell in particular begins to disappear. HC was seen as the least acceptable alternative for all groups, which was attributed to its high cost and potential to cause necrosis.

The effectiveness of an anesthetic agent depends on the active ingredients it contains (49). Since the current study focuses on the effect of a commercial anesthetic mixture on three different fish species, the content of anesthetic mixture will provide insight into possible components. In the evaluated anesthetic mixture primarily composed of monoterpenes, 1-propanol serves as the component that enhances water solubility, allowing for improved integration of the formulation in aqueous environments (50). Eugenol is the active ingredient of clove oil, which is frequently used as an anesthetic agent. On the other hand, linalool and linalyl acetate are the active ingredients of lavender essential oil. Clove oil has long been used both commercially and scientifically as an anesthetic for many species (35, 51–54). In general, it is an environmental friendly alternative to synthetic anesthetic agents, even though its use in high concentrations on fish may cause physiological effects. Lavender oil is an essential oil that has recently been shown to have an anesthetic effect on different species (12, 55, 56). Although both essential oils are important alternatives to synthetic agents, their physiological output on fish is likely to be reduced if used together. The current study provides a guide for comparison.

## 5 Conclusion

The effect of an anesthetic agent on fish depends on many parameters. The trophic level of the fish determines the concentration/effectiveness of the anesthetic agent. This study showed that, under current environmental conditions, Danube sturgeon is more resistant to a commercial anesthetic mixture than common carp and rainbow trout. However, this is not a direct comparison since the living conditions of these fish species differ under normal circumstances, and the possible anesthetic effect varies depending on both the current environmental parameters and fish biology (age, size, and weight). Under the present conditions, the effective concentration of the anesthetic mixture was higher for the Danube sturgeon. This means that sturgeon was exposed to higher anesthetic concentrations, which resulted in more pronounced histological damage. A lower anesthetic concentration was deemed unacceptable, as it significantly prolonged the induction time. In conclusion, the anesthetic concentration varies depending on the fish species. In this context, the best concentrations were determined by evaluating 10 different criteria using the PROMETHEE decision model. While these criteria are sufficient for assessing the suitability of an anesthetic agent, more detailed studies are needed to investigate stress indicators in fish. In future studies, different anesthetic agents can be compared using a multi-criteria decision-making model, and a combined anesthetic agent can be developed. In addition to synthetic substances, although natural essential oils are used individually, a mixture of different essential oils as an anesthetic agent is a topic worth investigating.

## Data Availability

The original contributions presented in the study are included in the article/supplementary material, further inquiries can be directed to the corresponding author.
